# Nipple-areola complex prosthesis as a new arsenal for breast reconstruction

**DOI:** 10.1016/j.jpra.2023.11.006

**Published:** 2023-11-20

**Authors:** Ali Modarressi, Louisa Empen, Charlotte Favre

**Affiliations:** a Plastic, reconstructive, and aesthetic surgery unit, Surgery department, University Hospitals of Geneva, Rue Gabrielle-Perret-Gentil 4, Geneva 1205, Switzerland; b Faculty of Medicine, University of Geneva, Switzerland; c Plastic, reconstructive & aesthetic center, Avenue de Champel 24, Geneva 1206, Switzerland

**Keywords:** Nipple-areola complex reconstruction, Breast reconstruction

## Abstract

Nipple and areola complex (NAC) recreating is primordial to achieve a complete breast reconstruction. Some patients would not benefit or choose to undergo surgical or tattoo NAC reconstruction. Recently, innovative NAC silicone prostheses have been developed that are applied over the skin and come in various shapes, sizes, and colors to match different NAC tones and sizes.

Our prospective study aims to evaluate for the first time, to our knowledge, the safety of these prostheses and patients’ and surgeons’ satisfaction using a 30-day survey. Twenty patients using these NAC prostheses after unilateral breast reconstruction postmastectomy with NAC excision were included.

All patients except one (95%) were satisfied/very satisfied with the aspect and symmetry of NAC prostheses compared with native contralateral NAC. All patients were more/much more satisfied with their body image and self-esteem using the NAC prostheses. Except for 2 (90%), no participant presented any skin reaction, infection, or erosion. Most patients (68.75%) used the prosthesis every day, and others—occasionally. All participants stated they would recommend these prostheses to other women as a temporary or definitive solution.

With these promising results demonstrating a high safety and satisfaction rate, we are confident that this simple, safe, noninvasive, and cost-effective device should be proposed to all patients to improve the management of breast reconstruction and offer body integrity to patients. Health insurance should consider reimbursement for these prostheses in patients after mastectomy or with NAC deformation after trauma, surgery, or congenital.

## Introduction

Breast cancer is the most frequent cancer in women. Although more conservative treatment is performed with a high survival rate, in some cases, mastectomy with resection of the nipple-areola complex (NAC) is, unfortunately, mandatory. It is recognized that, in women, the breast is a symbol of femininity, fertility, and maternity. The removal of one or both breasts profoundly breaks the patient's representation, self-esteem, and self-confidence. Breast reconstruction, including the reconstruction of the NAC after a mastectomy, is therefore fundamental. Different breast reconstruction techniques with restoration of the desired breast's shape and volume are possible. The breast reconstruction procedures have been showed to improve women's quality of life. However, after these procedures, women end up with a breast with an incomplete appearance due to the absence of NAC, which can impact their body perception. The NAC reconstruction is primordial for many patients. It has been shown that NAC reconstruction improves patient's body image, decreases their feeling of mutilation, and gives them the feeling of having overcome their disease.^1^ The NAC reconstruction is performed by surgical procedure or by tattoo in general. Both solutions are performed months after the breast reconstruction; therefore, patients must conform to an “incomplete” breast for a while. Moreover, surgical NAC reconstruction requires another surgery with another undesirable scar, and tattooed NAC does not offer a 3D nipple reconstruction, which could be annoying for patients when dressing with light bras.

An external silicone NAC prosthesis has been recently developed to address these disadvantages. This innovative nonsurgical approach is designed and produced by a patient, Michelle Kolath–Arbel, who was diagnosed with breast cancer and treated with a total mastectomy and NAC resection. She developed this prosthesis for women in situations similar to hers.

**Figure 1:**
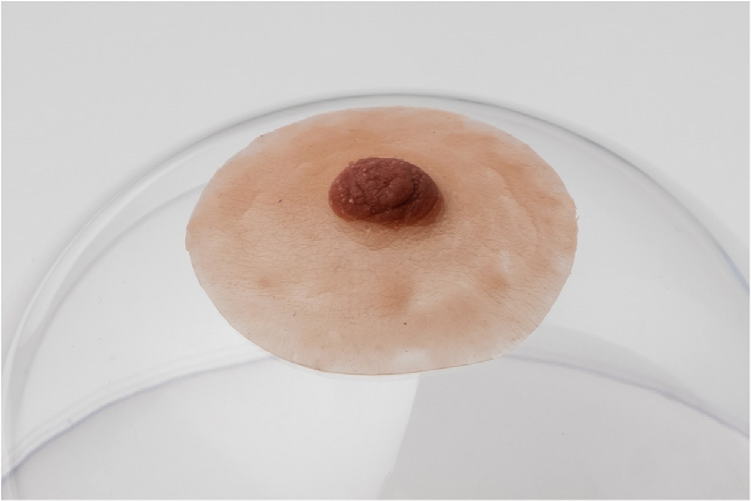
Example of a NAC prosthesis.

The purpose of these prostheses applied over the skin is to reproduce the appearance of a natural NAC (Figure 1). The patient can place it herself using a special medical glue and use it at any time. It remains on the skin for up to 2 weeks and is reusable. Its lifespan is estimated at 2 years with regular and meticulous maintenance. This prosthesis could be a temporary solution between mastectomy and the NAC reconstruction. If there are contraindications to NAC reconstruction or the patient's personal choice, it can also be considered a permanent solution. It offers a nonsurgical alternative, allowing to obtain the appearance of a complete breast quicker than other procedures.

This NAC prosthesis is a recent solution; no study has yet assessed its safety and efficacy. Thus, our prospective study aims to evaluate its safety, tolerability, and eventual side effects and to determine patient and surgeon satisfaction.

## Material and methods

NAC prosthesis was proposed to 20 consecutive patients. The inclusive criteria were female patients who underwent an immediate or delayed breast reconstruction (autologous or implant-based) after mastectomy with NAC excision. To be able to compare NAC prosthesis with native contralateral NAC, patients who underwent bilateral NAC excision were excluded. The NAC prosthesis was applied after wound healing was completed (e.g., around 1-month post-surgery). For patients requiring adjuvant radiotherapy, the application of NAC prosthesis was postponed until after the end of this treatment.

**Figure 2:**
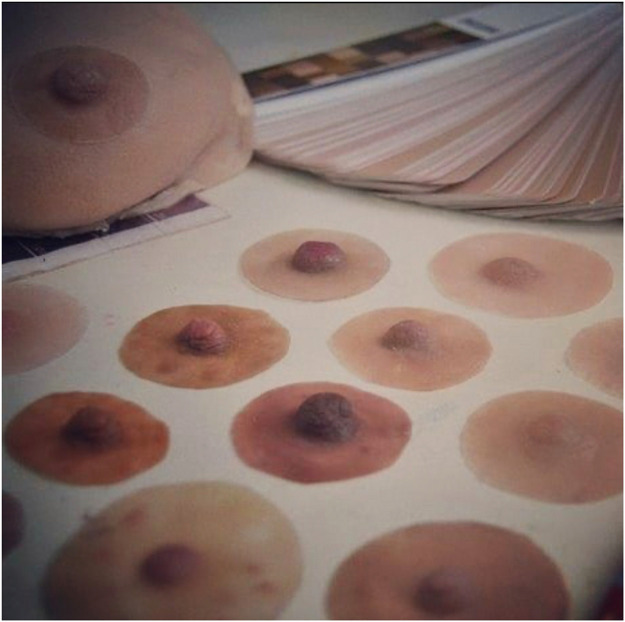
Different choices of prefabricated NAC prosthesis.

The NAC prostheses are hand-made by Pink Perfect (Israel) in silicone to reproduce the appearance and feel of the skin. It counts with 2 presentations developed in a similar way: prefabricated ready-to-wear or tailor-made prosthesis. In this study, prefabricated prostheses were used. They are available as 3 models, each with 9 different nuances: different colors, areola sizes, and nipple projection. (Figure 2) These external devices stick to the skin using a medical silicone-based glue composed of factor II dispersed in ethyl acetate. This glue is suitable for intense activities in the presence of moisture, sweating, or everyday life during variations in high temperatures. It has been tested for several security features, such as cytotoxicity, genotoxicity, pyrogenicity, skin irritation, and systemic toxicity. Each prosthesis is delivered with this specific glue and a user guide. The cost for each implant is 125 US$.

During the first visit, after having signed the consent form in agreement with local ethical committee approval, the patient chose the adequate NAC prosthesis among available ready-to-wear NAC prostheses comparing it with the contralateral native NAC. The patient received information on how to apply the NAC prosthesis and to answer a specific questionnaire.

To assess the NAC satisfaction particularly, we had to develop a specific questionnaire—one for patients and another for surgeons—based on BREAST-Q survey. The patient questionnaire assessed 5 different items: 1) demography, oncological, and breast reconstruction information; 2) aesthetic satisfaction—general appearance, color, texture, shape, dimension, relief, elasticity, finesse, details and symmetry; 3) practicability and circumstance of usage—how often and under which circumstances the participants wear the prosthesis, ease of using the prosthesis; 4) tolerance and biocompatibility: dermatological or systemic reactions such as erosions, erythema or infections; 5) effect on body image and self-image. The questionnaire was submitted during the first visit, and patients filled out the questionnaire on day 7 and day 30 and returned the questionnaires anonymously. The surgeon evaluated the same subjects as items 2) and 3) of the patient's questionnaire on day 7 and day 30.

## Results

The mean age of 20 patients included in the study was 54.15 years (31–77 years). All patients had undergone a unilateral mastectomy for cancer and breast reconstruction: 75% implant-based and 25% autologous reconstruction; 59.09% had an immediate breast reconstruction. Half of the patients had chemotherapy, and 40% received radiotherapy before applying the NAC prosthesis. The median time lapse between breast reconstruction and NAC application was 8 months (1 month-23 years).

**Figure 3:**
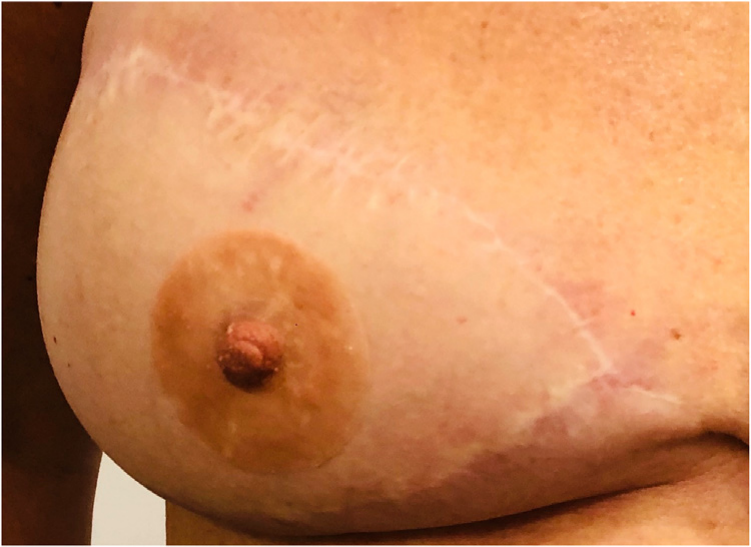
DIEP-based breast reconstruction with NAC prosthesis.

**Figure 4:**
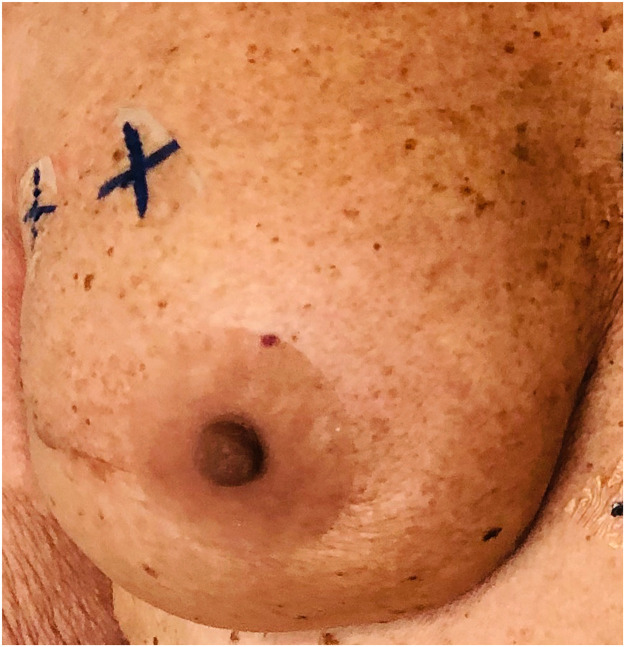
Implant-based breast reconstruction with NAC prosthesis before radiotherapy treatment.

**Figure 5:**
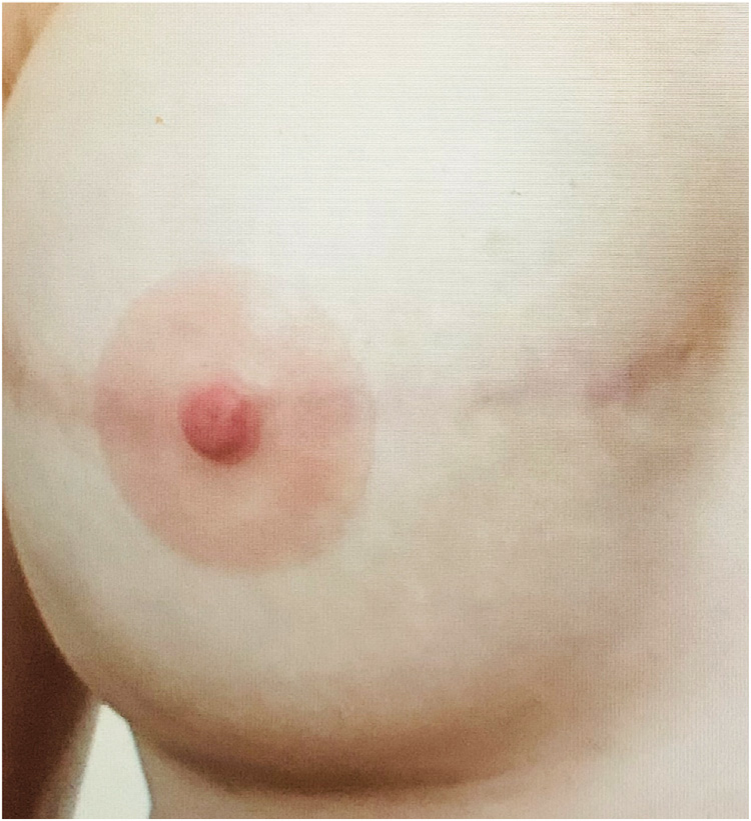
Implant-based breast reconstruction with NAC prosthesis.

All patients except one (95%) were satisfied or very satisfied with the prosthesis aspect, with an average score of 4.5/5. The highest satisfaction rate was with the color (100% satisfied). The thickness and texture were the lowest appreciated items, and their satisfaction rate was 75%, with an average of 4/5 (Figures 3, 4, and 5).

Regarding the effect of NAC prosthesis on body image and self-image, all patients are more or much more satisfied with their body image and when looking naked at themselves when wearing NAC prosthesis compared with not wearing it. Most of them found that they looked more attractive (79%) and more feminine (84%). The answer rate to the item of “satisfaction in front of partner” was 65%, but 85% of responding patients estimated that using the NAC prosthesis improves this concern.

For tolerance and biocompatibility, only 2 patients showed a skin reaction, e.g., redness with itching. Interestingly these 2 patients had adjuvant radiotherapy, and the NAC prosthesis was applied only one month after the end of radiotherapy sessions. These reactions were temporary, and they went away a few days after removing the NAC prosthesis. Most patients (90%) did not present any skin reaction, infection, or erosion at the NAC prosthesis location.

Concerning practicability and circumstance of usage, patients used the NAC prosthesis on average 5.8 days per week. Most patients (68.75%) used the prostheses daily and others only occasionally, mostly in sports activity, wearing a tight-fitting dress or working. On day 7 control, patients reported that, on average, they could wear the prosthesis 5.8 consecutive days without reapplying the glue. On day 30, the average time reported to use the prosthesis with the glue was 7.7 days, even having a shower, bath, or engaging in water sports activities. All patients estimated that the usage of NAC prosthesis was simple or very simple and practical, with an average score of 4.4/5. They were satisfied or very satisfied with the practicability of prosthesis use and its skin adherence, even during sports activities or in the water. They were less satisfied with it during intimate relations, where 22% of responding patients were not satisfied with the skin adherence of the NAC prosthesis (this item was answered by 45% of patients only).

At the end of the study, all participants reported they would recommend these prostheses to other women. All patients but one estimated that the NAC prosthesis meets completely their expectations. Of them, 35% planned to conserve the NAC prosthesis as a definitive, another 35% stated they would like to have a surgical NAC reconstruction in the future, and 30% were still undecided. Interestingly, 3 patients who did not plan to undergo an NAC reconstruction decided to have a surgical reconstruction after using the NAC prosthesis.

Scores for different items were comparable between day 7 and day 30 evaluations. The plastic surgeons scored the aesthetic aspect slightly better than the patients. They observed fewer dermatological reactions: only one patient showed a local skin reaction (redness and edema) on day 7 that improved on day 30.

The participants answered all item questions except those on intimate relations: “Skin adherence of NAC prosthesis during intimate relation” and “Satisfaction with NAC prosthesis use during relations with your partner,” where 55% and 35% did not respond, respectively.

## Discussion

Nowadays, breast reconstruction should be a part of breast cancer treatment. Moreover, NAC reconstruction is considered an important component for a successfully and satisfyingly reconstructed breast. Goh SCJ et al. showed that for 96% of patients who underwent breast reconstruction, NAC reconstruction was important.^1^

Among different NAC reconstruction methods today, patients were more satisfied with a surgical NAC reconstruction (88%) than tattoo (70%). The lack of nipple projection and discoloration of the tattoo were the main elements of dissatisfaction with these techniques.^2^ Our study demonstrated that NAC prostheses offer a high satisfaction rate (95%), particularly with color (100%) and nipple projection (85%). Furthermore, patients expressed higher satisfaction with the presence of NAC and improved body image when wearing the NAC prosthesis. Other studies have also demonstrated that the presence of the NAC contributes to patients' overall well-being.^3^^,^^4^

Some patients refuse to have a surgical NAC reconstruction because they do not want to have a new operation or a new scar on their body. NAC marking tattoo is a nonsurgical procedure, but not accepted by some patients because the nipple projection is important for them and cannot be achieved by the tattoo method. An NAC prosthesis could be an interesting solution for these patients because it offers a satisfying nipple projection without additional surgery or scarring. Among our patients, 35% planned to conserve the NAC prosthesis as a definitive NAC “reconstruction.” Some of them wanted to use it only occasionally, for example, when the presence of nipple projection is important, wearing tightly fitted, or sports or swim dresses. On the other hand, 3 patients who did not plan to undergo any NAC reconstruction at all, after having used the NAC prosthesis, decided to have a surgical reconstruction. This decision change was probably related to the aesthetic and body image improvement that NAC prosthesis offered them, exceeding their expectations.

Most patients would like to have a “complete” reconstructed breast, including the NAC. Still, they must wait at least 6 to 12 months after the last breast reconstruction procedure before undergoing a surgical or tattoo NAC reconstruction. For these patients, the NAC prosthesis could be an adequate temporary solution. At the end of the study, 35% of patients would like to have a surgical NAC reconstruction in the future.

Patients evaluated the prostheses as practical and simple to use. They tolerated the prosthesis and the glue for its application well. Except for 2 patients who had minor temporary skin reactions, there were no complications, and a few days after removing the prosthesis, the skin reaction resolved without any residual lesion. These skin reactions developed mostly in patients post radiotherapy, particularly when applied soon after radiotherapy completion. Therefore, it would be reasonable to recommend using an NAC prosthesis at least 3 months after the end of radiotherapy.

In this study, patients used only prefabricated ready-to-wear prosthesis, and they declared a very high satisfaction rate and would recommend these prostheses to other women. If tailor-made implants could be used for some patients, the satisfaction rate, particularly with the satisfaction of symmetry with the contralateral native NAC, would be even higher. Likewise, if the prosthesis was used on both breasts for patients with bilateral mastectomy, the satisfaction rate would be higher.

## Conclusion

To our knowledge, this study is the first clinical trial to evaluate the safety, satisfaction, and practicability of NAC prostheses for women who have had a mastectomy with NAC resection. The results showed a high satisfaction rate among patients and plastic surgeons. The NAC prosthesis is a noninvasive method of NAC reconstruction as a temporary or permanent solution. It could also be used with other indications than after mastectomy, i.e., NAC deformation after trauma, surgery, or congenital.

Sharing these promising results, we would like to contribute to the management of breast reconstruction and offer improvement of body integrity to patients affected by mastectomy. This simple, safe, noninvasive, cost-effective device should be recommended to all patients and considered for reimbursement by health insurance.
